# Supplementary material for: A comprehensive sulfate and DOM framework to assess methylmercury formation and risk in subtropical wetlands
Source: Nat Commun. 2025 May 7;16:4253. doi: 10.1038/s41467-025-59581-w (PMC12058987; doi:10.1038/s41467-025-59581-w)
Supplement: Supplementary file 1 — Supplementary Information [file 41467_2025_59581_MOESM1_ESM.pdf]

# Supplementary Information

to

# A Comprehensive Sulfate and DOM Framework to Assess Methylmercury Formation and Risk in Subtropical Wetlands

Brett A. Poulin[1*], Michael T. Tate[2], Sarah E. Janssen[2], George R. Aiken[3⊥], David P. Krabbenhoft[2]

[1]Department of Environmental Toxicology, University of California Davis, Davis, CA 95616, USA (current address)
[2]U.S. Geological Survey, Mercury Research Laboratory, Madison, Wisconsin 53726, United States
[3]U.S. Geological Survey, Water Mission Area, Boulder, Colorado 80303, United States

*Corresponding author. Tel: +1 530 754 2454. *Email address:* bapoulin@ucdavis.edu (B.A. Poulin),
[⊥]Deceased

Any use of trade, firm, or product names is for descriptive purposes only and does not imply endorsement by the U.S. Government. All data can be accessed in the corresponding data release (https://doi.org/10.5066/P976EGIX)[1]

Supplementary information includes: 12 Figures, 3 Tables, and 16 pages.

## Contents

Section S1. Unimodal Equation for Hg(II) Prediction ........ 2
Section S2. Quality Assurance and Quality Control on Mercury Measurements........ 2
Section S2. Supplemental Tables........ 4
Section S3. Supplemental Figures ........ 7
References........ 16

## Section S1. Unimodal Equation for Hg(II) Prediction

Relationships between surface water filtered inorganic divalent mercury (f.Hg(II)) concentration and sulfate ($SO_4^{2-}$) concentration were fit using a log normal 3 parameter equation (Equation S1)

$$f.Hg(II) = a \times e^{\left(-0.5 \times \frac{\left[\frac{\ln(SO_4^{2-}/x_0}{b}\right]^2}{SO_4^{2-}}\right)} \quad \text{(S1)}$$

where $x_0$, $a$, and $b$ were modeled using field measurements of $SO_4^{2-}$ and f.Hg(II) concentration, respectively (SigmaPlot v.14.5). $x_0$, $a$, and $b$ were 1723, 177.5, and 2.41 ($R^2$=0.18, $p$=0.09; $n$=93), respectively, for f.Hg(II).

## Section S2. Quality Assurance and Quality Control on Mercury Measurements

The following quality assurance and quality control checks were employed in this study for mercury measurements. First, field process blanks ($n$=15) were collected by filtering and analyzing ultrahigh purity water (≥18.2 MΩ cm) from the laboratory that was taken into the field and processed concurrent and identical to environmental field samples. Field process blanks were used to assess the potential for contamination of filtered or particulate samples for total mercury (f.HgT, p.THg) and methylmercury (f.MeHg, p.MeHg), and compared against environmental samples (**Figure S12**). The concentrations of all fractions of mercury species in process blanks were below or at the method detection limits (f.HgT, average of 0.05 ng/L; p.THg, average of 0.012 ng/L; f.MeHg, average of 0.003 ng/L; p.MeHg, average of 0.001 ng/L), as detailed in the associated data release[1] and shown in **Figure S12**. Approximately 5% of all field wetland samples were collected in replicate, and the concentrations of mercury fractions were within 5.5%.

Laboratory quality assurance and quality control checks were as follows. For all mercury measurements, analytical replicates, matrix spikes, and certified reference materials were used to assess analytical accuracy and precision. 10% of all analyses for f.HgT, f.MeHg, and p.HgT were matrix spike samples that had to achieve a recovery threshold range to pass. For filtered total mercury (f.HgT) and

particulate total mercury (p.HgT) measurements, all samples are run in at least analytical duplicate. For f.HgT measurement, if the relative percentage difference (RSD) was greater than 10% between duplicate analyses, then samples were analyzed in a triplicate and the RSD was confirmed to be <10%. If the RSD was ≥10%, the concentration was flagged in the associated data release.[1] For p.HgT measurements, analytical duplicates had to achieve an RSD of <10% and spike recoveries were within 85-115% to pass. With each batch of p.HgT analyses, CRM IAEA-456 (International Atomic Energy Agency; coastal sediment) was analyzed and had to achieve a recovery of 85-115%. For f.MeHg measurements, 10% of all samples were analyzed as analytical matrix spike duplicates; to pass, matrix spike duplicates had to be within 75-125% spike recovery and the absolute difference must be <25%. With each batch of f.MeHg analyses, CRM SQC1238 (Sigma Aldrich; methylmercury in sediment) was analyzed and 75-125% recovery was required to pass.

## Section S2. Supplemental Tables

**Table S1.** Site locations and distances from canals of sites samples in the Florida Everglades.

| Site ID | Distance from Canal (km)[a] | Longitude | Latitude |
|---|---|---|---|
| **Water Conservation Area 2A (WCA-2A)** | | | |
| WCA-2A – Canal (L-6) | -- | -80.47684 | 26.42693 |
| WCA-2A-P | 0.1 | -80.47643 | 26.42628 |
| WCA-2A-O | 0.2 | -80.47601 | 26.42506 |
| WCA-2A-N | 0.8 | -80.47415 | 26.41936 |
| WCA-2A-M | 1.6 | -80.47256 | 26.41225 |
| WCA-2A-L | 2.6 | -80.46967 | 26.40397 |
| WCA-2A-K | 3.6 | -80.46778 | 26.39494 |
| WCA-2A-J | 4.6 | -80.46429 | 26.38676 |
| WCA-2A-I | 5.7 | -80.46355 | 26.37650 |
| WCA-2A-H | 6.5 | -80.46056 | 26.37002 |
| WCA-2A-G | 7.7 | -80.45958 | 26.35972 |
| WCA-2A-F | 8.6 | -80.45748 | 26.35110 |
| WCA-2A-E | 10.0 | -80.45661 | 26.33913 |
| WCA-2A-D | 10.8 | -80.45321 | 26.33183 |
| WCA-2A-C | 12.1 | -80.44868 | 26.32129 |
| WCA-2A-B | 13.2 | -80.44582 | 26.31121 |
| WCA-2A-A | 14.4 | -80.44772 | 26.30093 |
| **Water Conservation Area 3A (WCA-3A)** | | | |
| L-28 Canal | -- | -80.82810 | 26.16678 |
| WCA-3A-O | 1.3 | -80.80122 | 26.09357 |
| WCA-3A-N | 4.1 | -80.78688 | 26.07145 |
| WCA-3A-M | 7.0 | -80.77201 | 26.04907 |
| WCA-3A-L | 9.3 | -80.76062 | 26.03111 |
| WCA-3A-K | 11.9 | -80.74679 | 26.01058 |
| WCA-3A-J | 15.0 | -80.73361 | 25.98554 |
| WCA-3A-I | 17.8 | -80.72416 | 25.96166 |
| WCA-3A-H | 20.5 | -80.71834 | 25.93772 |
| WCA-3A-G | 23.5 | -80.71089 | 25.91117 |
| WCA-3A-F | 26.0 | -80.70715 | 25.88864 |
| WCA-3A-E | 28.9 | -80.70461 | 25.86163 |
| WCA-3A-D | 31.8 | -80.70169 | 25.83538 |
| WCA-3A-C | 34.6 | -80.70033 | 25.81032 |
| WCA-3A-B | 37.3 | -80.69942 | 25.78540 |
| WCA-3A-A | 39.7 | -80.69696 | 25.76387 |
| **Loxahatchee National Wildlife Refuge (WCA-1)** | | | |
| LOX-139 | 0.10 | -80.315611 | 26.62324 |
| LOX-137 | 1.04 | -80.321750 | 26.61501 |
| LOX-136 | 3.96 | -80.337883 | 26.59293 |
| LOX-5 | 8.36 | -80.35794 | 26.55727 |
| LOX-8 | 12.5 | -80.33791 | 26.52398 |

*a* Distances for sites in WCA-2A are from the L-6 canal, in WCA-3A are from the L-28 canal, and in LOX are from the Palm Beach Canal.

**Table S2.** Sampling information for each of the Water Conservation Area (WCA) and Arthur R. Marshall Loxahatchee National Wildlife Refuge (LOX) transects, including date(s) sampled and the sampling of surface water (SW) and/or pore water (PW), cumulative precipitation and atmospheric deposition of total Hg for the three months prior to sampling, and maximum air temperature at the time of sampling.

| | **WCA-2A Transect** | | | **WCA-3A Transect** | | | **LOX Transect** | | | **Cum. Precipitation, Annual Total (3 months prior to sampling)**[b] (cm) | **Cum. Atm. Hg Deposition, Annual Total (3 months prior)**[b] (μg/m²) | **Daily Max./Min. Air Temp (°C) Range at Sampling**[c] |
|---|---|---|---|---|---|---|---|---|---|---|---|---|
| **Year** | **Date** | **SW** | **PW** | **Date** | **SW** | **PW** | **Date** | **SW** | **PW** | | | |
| 2012 | 07/17/2012 | X | -- | -- | -- | -- | -- | -- | -- | 159 (62.5) | 21.5 (8.87) | 31.7 / 22.2 |
| 2013 | 05/07/2013 | X | -- | 05/08/2013 | X | -- | -- | -- | -- | 148 (27.9) | 20.6 (2.71) | 32.2-32.8 / 14.4-16.7 |
| 2014 | 08/19/2014 | X | X | 8/20/2014 | X | X | -- | -- | -- | 141 (56.5) | 22.1 (12.3) | 36.1 / 23.9-24.4 |
| 2015 | 11/17/2015 | X[a] | X[a] | 11/18/2015 | X[a] | X[a] | 11/16/2015 | X | X | 102 (35.4) | -- (5.32) | 26.1-29.4 / 21.1-23.9 |
| 2016 | -- | -- | -- | -- | -- | -- | -- | -- | -- | 138 (--) | 18.3 (--) | -- |
| 2017 | 08/15/2017 | X[a] | X[a] | 8/16/2017 | X[a] | X[a] | -- | -- | -- | 173 (66.4) | 17.7 (9.40) | 34.4-37.8 / 23.3-23.9 |
| 2018 | 07/17/2018 | X[a] | X[a] | 7/18/2018 | X[a] | X[a] | -- | -- | -- | 100 (59.4) | 13.1 (6.08) | 33.9 / 23.3 |
| 2019 | 12/02/2019 | X[a] | X[a] | 12/03/2019 | X[a] | X[a] | 12/02/2019 | X[a] | X[a] | 120 (25.9) | 14.1 (2.04) | 21.7 / 7.8 |
| 1997-2023 | | | | | | | | | | 128 ± 32.7 | 17.3 ± 4.41 | |

[a] Identifies the sampling of a truncated transect (see associated data release).[1]
[b]Data are from the National Atmospheric Deposition Program, Mercury Deposition Network.[2] Data from 2012-2014 were from Site FL34 and from 2015-2019 were from Site FL95, both located within the Water Conservation Areas.
[c]Data from the Royal Palm Ranger Station, maintained by the National Oceanographic and Atmospheric Administration (NOAA).[3]

**Table S3**. Water chemistry data of canals that discharge into Water Conservation Area 2A (WCA-2A) and 3A (WCA-3A) near the top of the hydrologic transects, and the Palm Beach canal that contributes to the perimeter around the Arthur R. Marshall Loxahatchee National Wildlife Refuge (LOX). Measurements include filtered and particulate inorganic Hg(II) (f.Hg(II), p.Hg(II)), filtered and particulate methylmercury (f.MeHg, p.MeHg), percent f.MeHg, dissolved organic carbon (DOC), dissolved organic matter (DOM) ultraviolet absorbance at 254 nm ($UV_{254}$), DOM specific ultraviolet absorbance at 254 nm ($SUVA_{254}$), sulfate ($SO_4^{2-}$), and chloride ($Cl^-$). Mean values ± 1 standard deviation are presented. Values in parentheses indicate the number of discrete samples. All data can be found in the corresponding data release.[1]

| Water Parameter | L-6 and Hillsboro Canals (WCA-2A) | L-28 Canal (WCA-3A) | Palm Beach Canal at E Canal Street (LOX) |
|---|---|---|---|
| f.Hg(II) (ng/L) | 0.67±0.20 (*n*=11) | 0.71±0.26[a] (*n*=8) | 0.74±0.21 (*n*=4) |
| p.Hg(II) (ng/L) | 0.36±0.31 (*n*=8) | 0.43±0.14[a] (n=5) | 4.04±5.28 (*n*=4) |
| f.MeHg (ng/L) | 0.07±0.04 (*n*=11) | 0.09±0.07[a] (*n*=7) | 0.10±0.07 (*n*=4) |
| p.MeHg (ng/L) | 0.02±0.01 (*n*=8) | 0.17±0.31[a] (*n*=6) | 0.18±0.15 (*n*=4) |
| % f.MeHg | 9.4±4.2 (*n*=11) | 10.8±4.5 (*n*=7) | 10.3±6.4 (*n*=4) |
| DOC (mg/L) | 33.1±4.2 (*n*=7) | 19.6±2.9 (*n*=8) | 19.9±12.5 (*n*=4) |
| DOM $UV_{254}$ (AU/cm) | 1.15±0.20 (*n*=7) | 0.64±0.11 (*n*=8) | 0.58±0.54 (*n*=4) |
| DOM $SUVA_{254}$ (L/mg m) | 3.48 ± 0.16 (*n*=7) | 3.30±0.34 (*n*=8) | 2.63±0.65 (*n*=4) |
| $SO_4^{2-}$ (mg/L) | 64.7±32.4 (*n*=8) | 6.84±3.65 (*n*=8) | 43.9±40.6 (*n*=7) |
| $Cl^-$ (mg/L) | 131±37.4 (*n*=8) | 32.8±18.0 (*n*=8) | 96.2±58.7 (*n*=7) |

[a]Summary data omits an outlier measured on 8/18/2014 (f.Hg(II) = 2.72 ng/L; p.Hg(II) = 0.55 ng/L; f.MeHg = 1.11 ng/L; p.MeHg = 0.14 ng/L)

## Section S3. Supplemental Figures

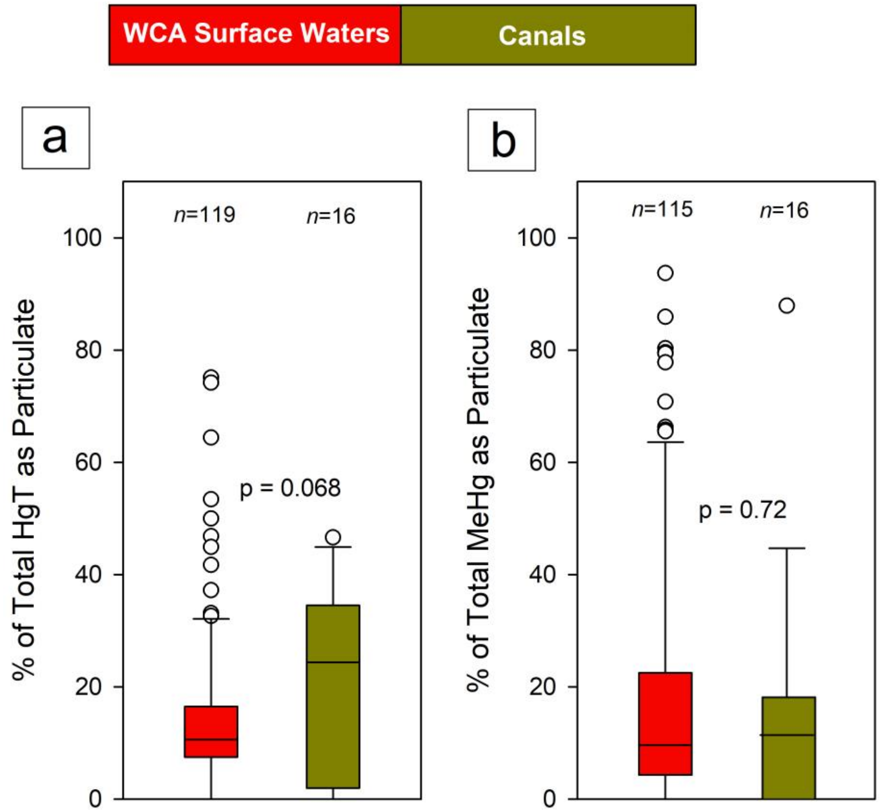


**Figure S1**. Box plots present median and quartile ranges of percentage of (a) total total Hg as particulate HgT and (b) total MeHg as particulate MeHg between surface waters of Water Conservations Areas (WCA-2A, 3A, LOX) and canals that drain into these wetlands (L-6 and L-28 canals). Error bars represent 10-90% percentiles, outliers are shown as data points, and p-values present results from a paired t-tests between WCA and canal data.

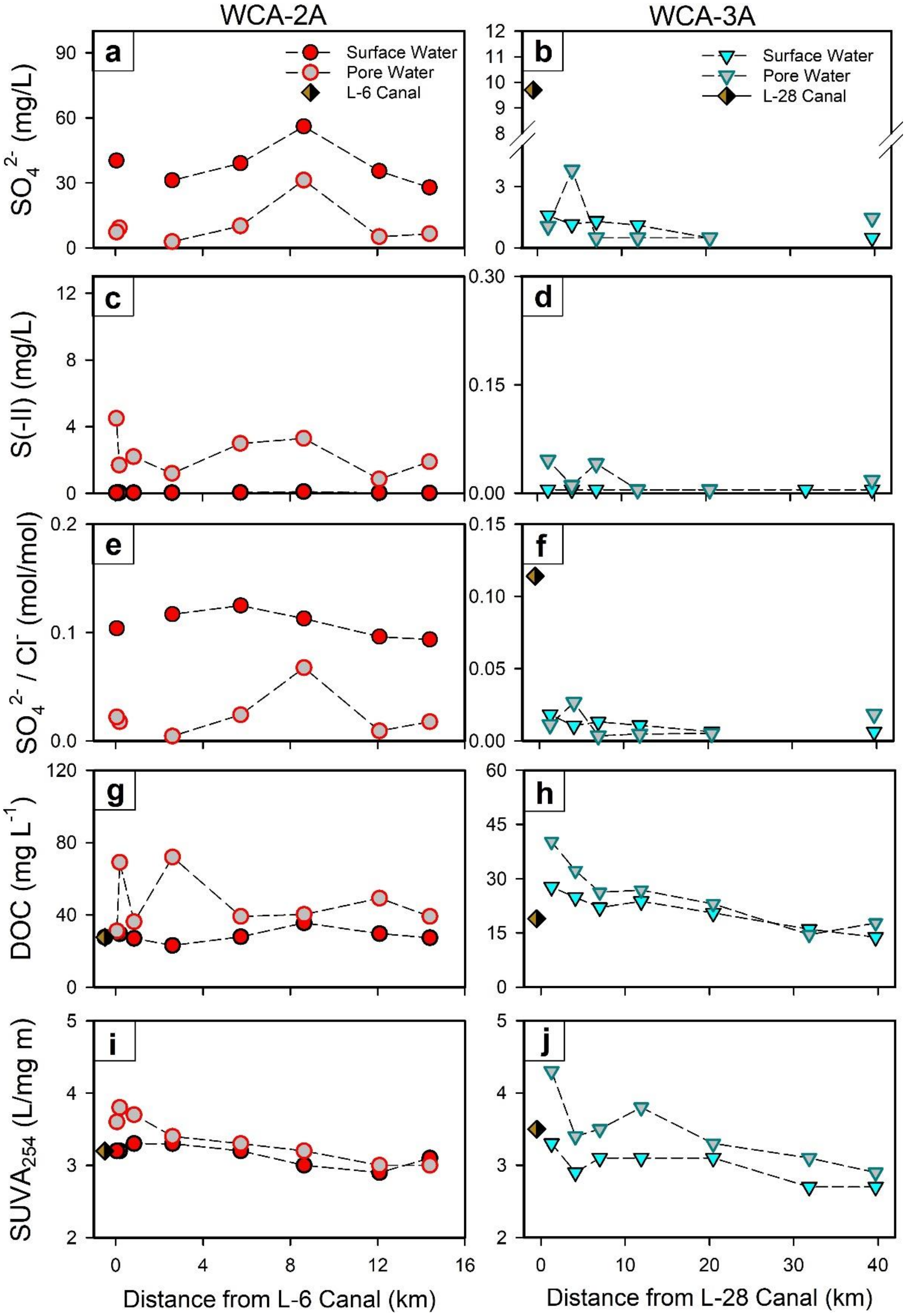


**Figure S2**. Biogeochemical constituents of (**a-b**) sulfate ($SO_4^{2-}$) concentration, (**c-d**) inorganic sulfide (S(-II)) concentration, (**e-f**) the molar ratio of $SO_4^{2-}$ to chloride ($Cl^-$), (**g-h**) dissolved organic carbon (DOC) concentration, and (**i-j**) dissolved organic matter (DOM) specific ultraviolet absorbance at 254 nm ($SUVA_{254}$) in Water Conservation Area 2A (WCA-2A) and WCA-3A as a function of distance from canal inputs (November 2015). For each transect, canal conditions are presented at the time of wetland sampling. Dashed lines are presented to guide the eye.

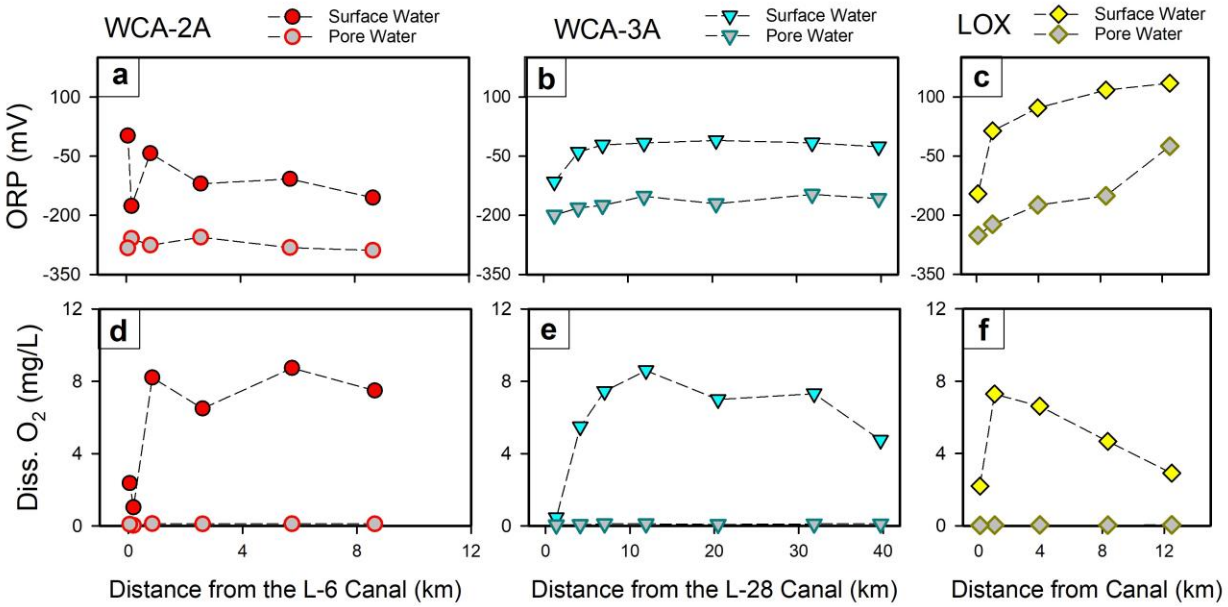


**Figure S3**. Measurements of (**a-c**) oxidation-reduction potential (ORP) and (**d-f**) dissolved oxygen ($O_2$) of surface and pore waters from Water Conservation Area 2A (WCA-2A) (August, 2014), WCA-3A (August, 2014), and the Arthur R. Marshall Loxahatchee National Wildlife Refuge (LOX) as a function of distance from canal inputs (November, 2015). Dashed lines are presented to guide the eye.

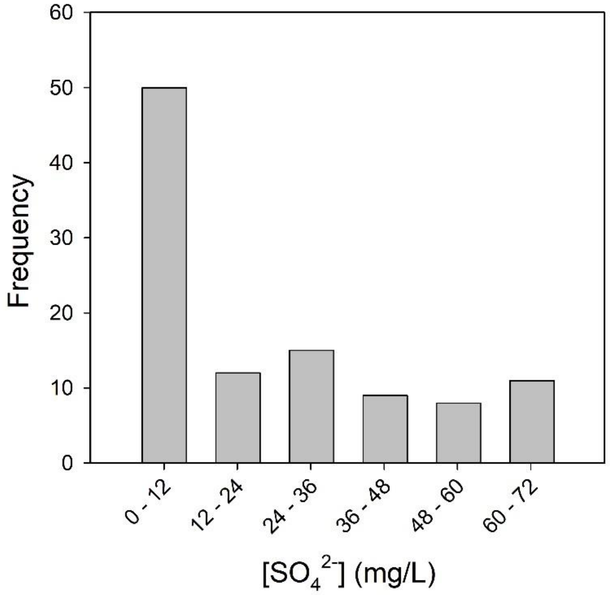


**Figure S4**. A histogram sulfate ($SO_4^{2-}$) concentrations in the three wetlands of the study, with sampling emphasis on low-to-intermediate concentrations (0-12 mg/L) and an equal distribution of samples at intermediate-to-high $SO_4^{2-}$ concentration (12-72 mg/L).

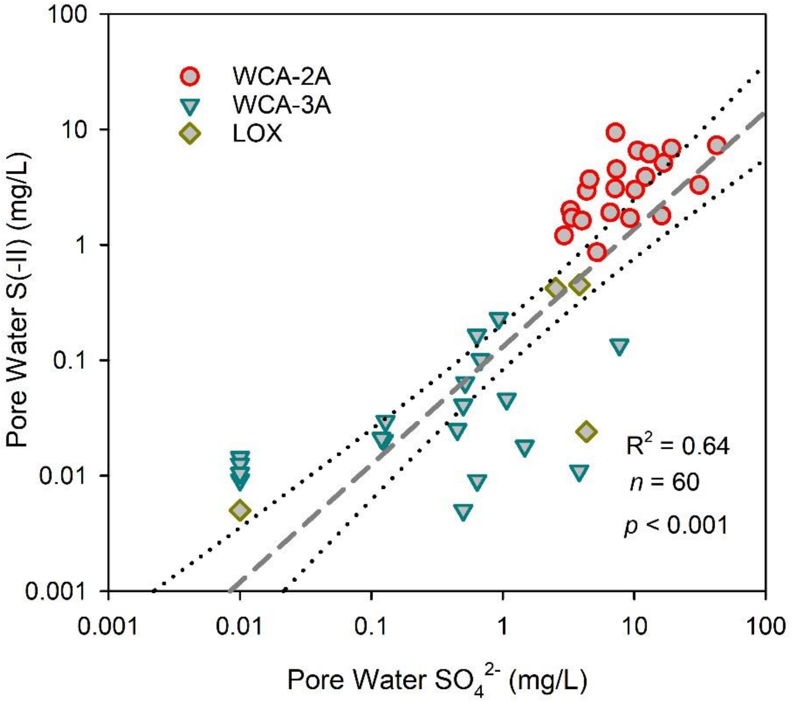


**Figure S5**. Scatter plots presenting linear correlations between the pore water concentrations of sulfate ($SO_4^{2-}$) and sulfide (S(–II)) across Water Conservations Areas (WCA-2A, 3A) and Arthur R. Marshall Loxahatchee National Wildlife Refuge (LOX). The dashed gray line and dotted blank lines present the linear fit and 95% confidence intervals of the fit, respectively.

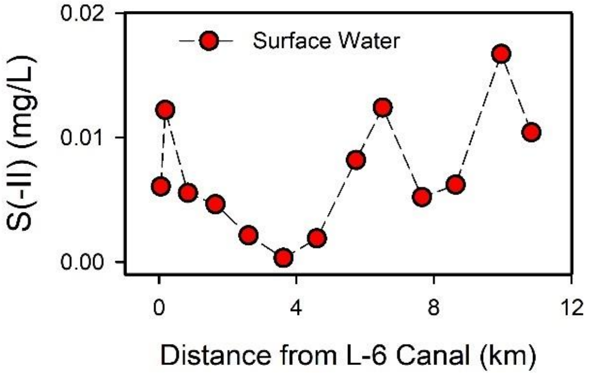


**Figure S6**. Inorganic sulfide (S(-II)) concentration in the surface water of Water Conservation Area 2A (WCA-2A) as a function of distance from canal inputs (August, 2014). Dashed lines are presented to guide the eye.

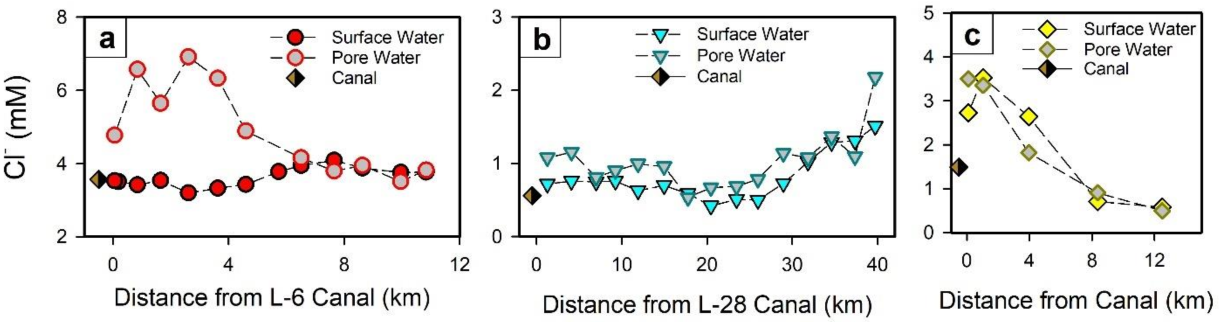


**Figure S7**. Chloride ($Cl^{-}$) concentration (**a**) Water Conservation Area 2A (WCA-2A; August, 2014), (**b**) WCA-3A (August, 2014), and (**c**) the Arthur R. Marshall Loxahatchee National Wildlife Refuge (LOX; November, 2015) as a function of distance from canal inputs. For each transect, canal conditions are presented at the time of wetland sampling. Dashed lines are presented to guide the eye.

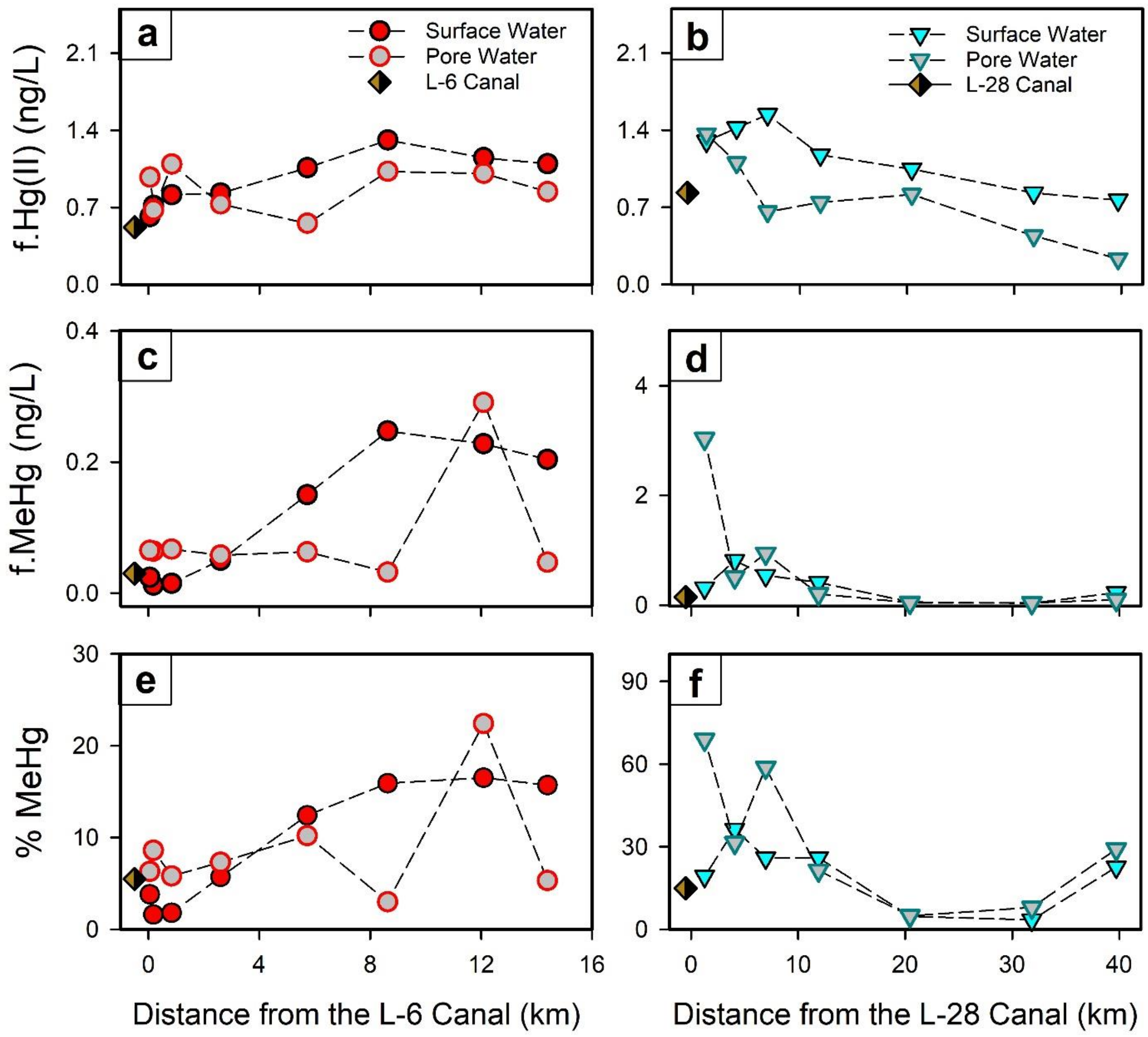


**Figure S8**. Concentrations of filtered (**a-b**) inorganic Hg (f.Hg(II)), (**c-d**) methylmercury (f.MeHg), and (**e-f**) the percentage of total Hg as MeHg (%MeHg) in Water Conservation Area 2A (WCA-2A) and WCA-3A as a function of distance from canal inputs (November 2015). For each transect, canal conditions are presented at the time of wetland sampling. Dashed lines are presented to guide the eye.

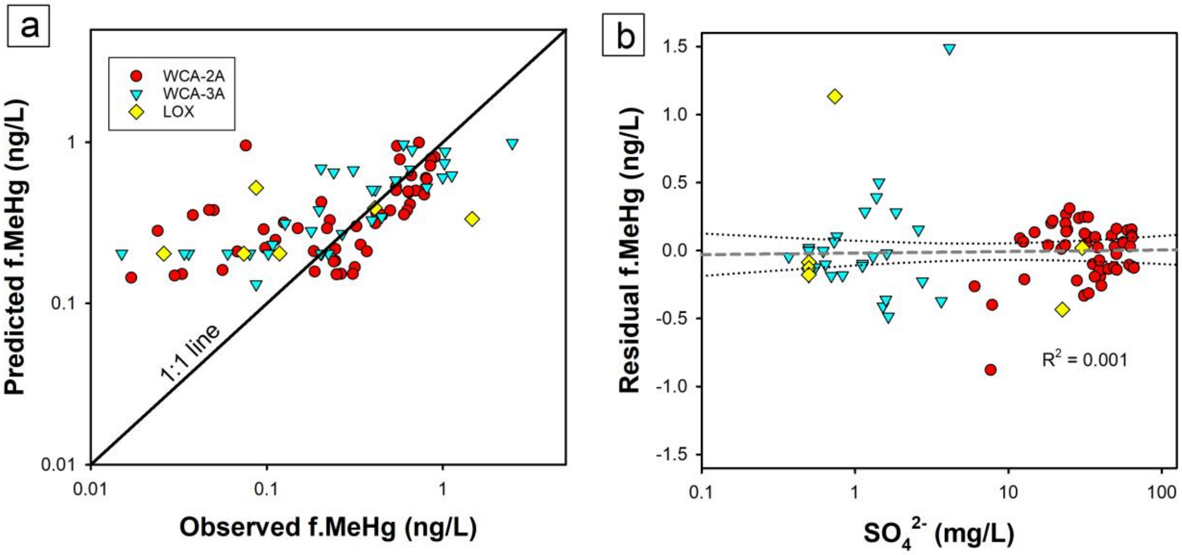


**Figure S9**. Scatter plots between (a) observed versus predicted filtered methylmercury concentration (f.MeHg) from the nonlinear regression (Equation 1, main text) and (b) residual f.MeHg versus sulfate ($SO_4^{2-}$) concentration across Water Conservations Areas (WCA-2A, 3A) and Arthur R. Marshall Loxahatchee National Wildlife Refuge(LOX). In subplot b the dashed gray line and dotted blank lines present the linear regression and 95% confidence intervals of the fit, respectively.

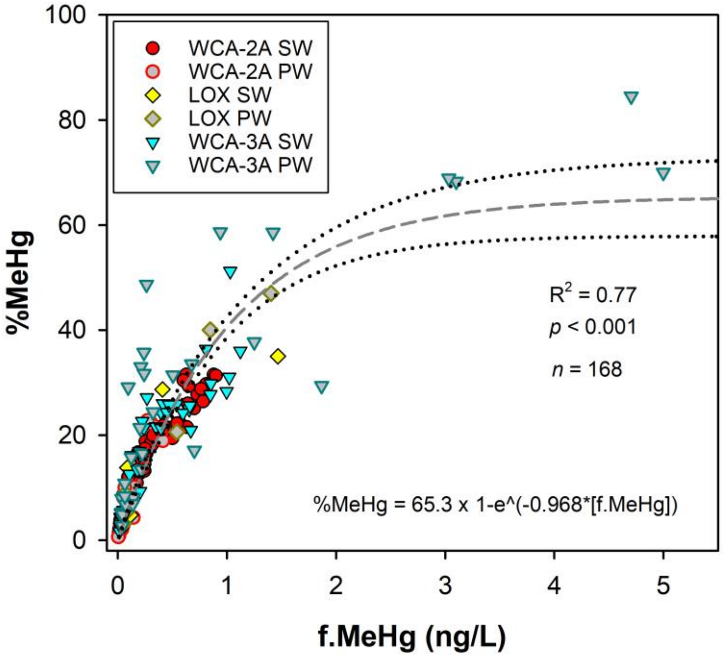


**Figure S10**. Scatter plot between the concentration of filtered methylmercury (f.MeHg) and the percentage of total mercury present as MeHg (%MeHg) for surface waters and pore waters across Water Conservations Areas (WCA-2A, 3A) and Arthur R. Marshall Loxahatchee National Wildlife Refuge(LOX). The dashed gray line and dotted blank lines present the exponential curve and 95% confidence intervals of the fit, respectively.

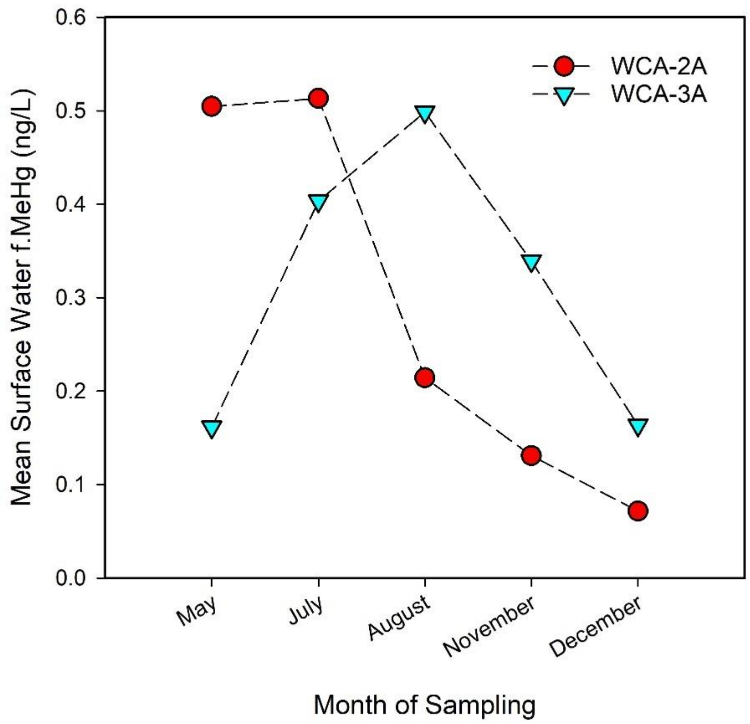


**Figure S11**. Plot of mean surface water filtered methylmercury (f.MeHg) concentration of water conservation area 2A (WCA-2A) and 3A (WCA-3A) over the seven sampling trips that spanned from May to December.

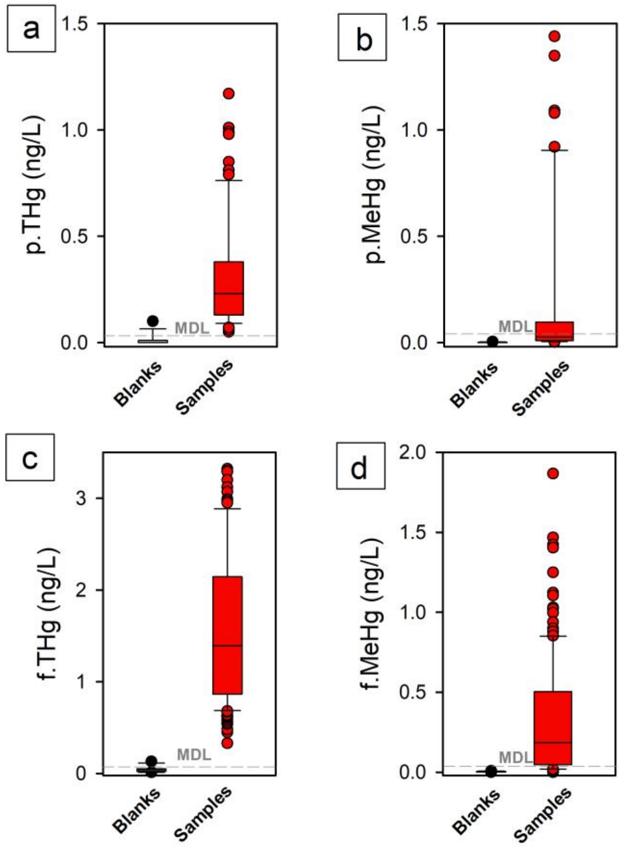


**Figure S12**. A comparison of concentrations of field process blanks (*n*=15) and environmental samples from the study wetlands including (a) particulate total Hg (p.THg) (*n*=126), (b) particulate methylmercury (p.MeHg) (*n*=126), (c) filtered total Hg (f.THg) (*n*=197), and (d) filtered methylmercury (f.MeHg) (*n*=197). The horizonal dashed gray line presents the method detection limit.

## Supplementary References


1. Tate, M. T. et al. Chemical Characterization of Water, Sediments, and Fish from Water Conservation Areas and Canals of the Florida Everglades (USA), 2012 to 2019. *U.S. Geological Survey Data Release*. https://doi.org/10.5066/P976EGIX. (2023)
2. *National Atmospheric Deposition Network (NADP), Annual Mercury Deposition Network Maps, 1998-2023*. NADP Program Office, Wisconsin State Laboratory of Hygiene. https://nadp.slh.wisc.edu/networks/mercury-deposition-network/ (Accessed 10/30/2024).
3. National Climatic Data Center (NCDC) NOAA. *NOAA Daily Surface Meteorologic Data at NCDC Royal Palm Ranger Station (ID-087760)(FCE LTER), South Florida, USA, May 1949 - Ongoing. Environmental Data Initiative.* (2022).
