# Supplementary material for: A comprehensive sulfate and DOM framework to assess methylmercury formation and risk in subtropical wetlands
Source: Nat Commun. 2025 May 7;16:4253. doi: 10.1038/s41467-025-59581-w (PMC12058987; doi:10.1038/s41467-025-59581-w)
Supplement: Supplementary file 3 — Reporting Summary [file 41467_2025_59581_MOESM3_ESM.pdf]

nature portfolio

Corresponding author(s): Brett Poulin

Last updated by author(s): Apr 18, 2025

Reporting Summary

Nature Portfolio wishes to improve the reproducibility of the work that we publish. This form provides structure for consistency and transparency in reporting. For further information on Nature Portfolio policies, see our Editorial Policies and the Editorial Policy Checklist.

Statistics

For all statistical analyses, confirm that the following items are present in the figure legend, table legend, main text, or Methods section.

n/a	Confirmed	
☐	☒	The exact sample size (*n*) for each experimental group/condition, given as a discrete number and unit of measurement
☐	☒	A statement on whether measurements were taken from distinct samples or whether the same sample was measured repeatedly
☐	☒	The statistical test(s) used AND whether they are one- or two-sided<br>*Only common tests should be described solely by name; describe more complex techniques in the Methods section.*
☒	☐	A description of all covariates tested
☐	☒	A description of any assumptions or corrections, such as tests of normality and adjustment for multiple comparisons
☐	☒	A full description of the statistical parameters including central tendency (e.g. means) or other basic estimates (e.g. regression coefficient) AND variation (e.g. standard deviation) or associated estimates of uncertainty (e.g. confidence intervals)
☐	☒	For null hypothesis testing, the test statistic (e.g. *F*, *t*, *r*) with confidence intervals, effect sizes, degrees of freedom and *P* value noted<br>*Give P values as exact values whenever suitable.*
☒	☐	For Bayesian analysis, information on the choice of priors and Markov chain Monte Carlo settings
☒	☐	For hierarchical and complex designs, identification of the appropriate level for tests and full reporting of outcomes
☒	☐	Estimates of effect sizes (e.g. Cohen's *d*, Pearson's *r*), indicating how they were calculated

Our web collection on statistics for biologists contains articles on many of the points above.

Software and code

Policy information about availability of computer code

Data collection	No software was used.
Data analysis	SigmaPlot V14.5 was used for statistical tests and fitting of data.

For manuscripts utilizing custom algorithms or software that are central to the research but not yet described in published literature, software must be made available to editors and reviewers. We strongly encourage code deposition in a community repository (e.g. GitHub). See the Nature Portfolio guidelines for submitting code & software for further information.

Data

Policy information about availability of data

All manuscripts must include a data availability statement. This statement should provide the following information, where applicable:

- Accession codes, unique identifiers, or web links for publicly available datasets
- A description of any restrictions on data availability
- For clinical datasets or third party data, please ensure that the statement adheres to our policy

All data can be accessed in the corresponding data release (https://doi.org/10.5066/P976EGIX).

Research involving human participants, their data, or biological material

Policy information about studies with human participants or human data. See also policy information about sex, gender (identity/presentation), and sexual orientation and race, ethnicity and racism.

Reporting on sex and gender	N/A
Reporting on race, ethnicity, or other socially relevant groupings	N/A
Population characteristics	N/A
Recruitment	N/A
Ethics oversight	N/A

Note that full information on the approval of the study protocol must also be provided in the manuscript.

Field-specific reporting

Please select the one below that is the best fit for your research. If you are not sure, read the appropriate sections before making your selection.

☐ Life sciences ☐ Behavioural & social sciences ☒ Ecological, evolutionary & environmental sciences

For a reference copy of the document with all sections, see nature.com/documents/nr-reporting-summary-flat.pdf

Ecological, evolutionary & environmental sciences study design

All studies must disclose on these points even when the disclosure is negative.

Study description	A series of field campaigns were conducted along existing hydrologic transects in three differing wetlands of the freshwater Florida Everglades. Surface water, pore water and fish were collected, and linkages were established between water quality metrics and the formation of methylmercury.
Research sample	Water sampled were analyzed for routine and advanced constituents that influence the cycling of mercury. Methylmercury concentrations in prey fish (Gambusia holbrooki) were used as an indicator of the availability of methylmercury to the aquatic food web.
Sampling strategy	Waters and fish were collected across hydrologic gradients and that span geochemical gradients across three freshwater, sawgrass dominated wetlands of the Florida Everglades.
Data collection	Field crew members recorded meta data of field samples (location, date, time, depth), which was included in the data release product.
Timing and spatial scale	Work was completed between 2012-2019. The spatial scale is limited to Water Conservation Areas 2A and 3A (WCA-2A, WCA-3A), and the Arthur R. Marshall Loxahatchee National Wildlife Refuge (LOX). All GPS coordinates are provided in the data release and SI of the paper.
Data exclusions	N/A
Reproducibility	Field replication was conducted over the study period. The relative percent deviation for water constituents is reported in the main text of the manuscript, and did not exceed 5.5%.
Randomization	N/A
Blinding	N/A

Did the study involve field work? ☒ Yes ☐ No

Field work, collection and transport

Field conditions	Water depth and flow conditions were obtained by the GrAdient-Related Discharge in an Everglades Network (GARDEN) model.
Location	All site latitudes and longitudes are provided in the associated data release product and in the SI (Table S1)
Access & import/export	Permits for field sampling were obtained by the South Florida Water Management District and the Arthur R. Marshall Loxahatchee

	National Wildlife Refuge
Disturbance	Minimal disturbances were made. The only minor disturbance was the insertion of the sipper (2.5" diameter) to a maximum depth of 10 cm for collection of pore water samples.

Reporting for specific materials, systems and methods

We require information from authors about some types of materials, experimental systems and methods used in many studies. Here, indicate whether each material, system or method listed is relevant to your study. If you are not sure if a list item applies to your research, read the appropriate section before selecting a response.

Materials & experimental systems

n/a	Involved in the study
☒	☐ Antibodies
☒	☐ Eukaryotic cell lines
☒	☐ Palaeontology and archaeology
☒	☐ Animals and other organisms
☒	☐ Clinical data
☒	☐ Dual use research of concern
☒	☐ Plants

Methods

n/a	Involved in the study
☒	☐ ChIP-seq
☒	☐ Flow cytometry
☒	☐ MRI-based neuroimaging

Plants

Seed stocks	N/A
Novel plant genotypes	N/A
Authentication	N/A
